# Detection of complex deletions in chromosomes 13 and 21 in a fetus by noninvasive prenatal testing

**DOI:** 10.1186/s13039-016-0213-4

**Published:** 2016-01-12

**Authors:** Ting Wang, Chengying Duan, Cong Shen, Jingjing Xiang, Quanze He, Jie Ding, Ping Wen, Qin Zhang, Wei Wang, Minjuan Liu, Hong Li, Haibo Li, Lili Zhang

**Affiliations:** Center for Reproduction and Genetics, Nanjing Medical University Affiliated Suzhou Hospital, Suzhou, Jiangsu 215002 China; Center for Medical Ultrasound, Nanjing Medical University Affiliated Suzhou Hospital, Suzhou, Jiangsu China

**Keywords:** Noninvasive prenatal testing, Karyotype analysis, Array-based comparative genomic hybridization, Subchromosomal abnormalities

## Abstract

**Background:**

To detect complex fetal subchromosomal abnormalities by noninvasive prenatal testing (NIPT).

**Case presentation:**

After routine prenatal serum screening, the plasma of high-risk pregnant women were tested via NIPT, and the NIPT results were further validated by fetal karyotype analysis and array-based comparative genomic hybridization (aCGH) through amniocentesis. In addition, the chromosome karyotypes of the parents were also analyzed. NIPT results indicated subchromosomal abnormalities in chromosomes 13 and 21; aCGH results showed 22 Mb and 16 Mb deletions in 13 q31.3 - q34 and 21q11.1 - q21.3, respectively; and the fetal karyotype was 45,XX, der(13),-21. The maternal karyotype 46,XX,inv(9)(p12q13),t(13;21)(q31.3;q21.3) was abnormal, while the paternal karyotype showed no obvious abnormality.

**Conclusion:**

In this study, we successfully detected complex deletions in chromosomes 13 and 21 in a fetus using NIPT, and NIPT can provide effective genetic information for the detection of fetal subchromosomal abnormalities.

## Background

In recent years, based on the high-throughput sequencing technology, noninvasive prenatal testing (NIPT) has been shown to exhibit a strong technical advantage [1–3]. In China, NIPT has acquired technical access from health sector and earned widespread clinical application. Additionally, more and more studies indicated NIPT could effectively detect fetal subchromosomal abnormalities and microdeletions/ microduplications, which will expand its clinical application spectrum, provide more testing information and show great potential applications [4–6].

In this study, a rare complex deletions in chromosomes 13 and 21 in a fetus was detected among 15,000 patients with high risk of serum screening and further validated by systematic cellular and molecular genetic analysis in our center. We will discuss this case in detail in this paper to further reveal the value of NIPT.

## Case presentation

The patient was a 29-year-old mother (“gravida 4, para 1”, G4P1). The patient had four pregnancies, and she delivered a healthy girl three years ago. She also had a spontaneous abortion and two induced abortions. She missed the first trimester screening, and serum screening in her second trimester of pregnancy in another hospital suggested that her baby was at high risk of Down syndrome (1/110). Then she was transferred to our center, and she opted for NIPT to avoid potential complications of invasive testing at 19 weeks of gestation.

## Methods

### Noninvasive prenatal testing (NIPT)

Experiments and data analysis were performed through the technology platform provided by DaAn Gene limited company. 10 mL peripheral blood was extracted from the pregnant women with EDTA anticoagulation, and cell-free DNA was extracted from the plasma by high speed centrifugation. After the DNA library was prepared and sequenced, the risk rate of chromosome abnormality (aneuploidy, chromosome copy number variation etc.) was calculated via bioinformatics analysis. Briefly, chromosomes were divided into more than 3000 windows (10^6^ as a unit), and 200 NIPT negative cases were selected as a control. Reads number of each window was calculated according to the position of the free DNA in the chromosome after alignment of short reads into the reference human genome (hg19). The mean and variance of reads number from each window of the 200 samples were calculated respectively. Based on this, deletions in each window of actual sample were calculated using the following formula: Z score = Actual reads number - Mean of reads number/Variance of reads number. For further detection of fetal large chromosomal deletions/duplications, the methods reported by Chen et al. was adopted, which was proved to be powerful to detect fetal chromosomal deletions/duplications of > 10 Mb using low coverage whole genome sequencing of maternal plasma [7].

### Chromosome karyotype analysis

Under sterile conditions, the amniocentesis was performed with the guidance of ultrasound. The amniotic fluid was centrifuged, inoculated in the culture medium and cultured under 37 °C. Once many circular translucent dividing cells were emerged, colchicine was added and cultured for another three hours. When the number of circular translucent cells increased, cells were harvested for chromosome preparation. According to the principle of “An International System for Human Cytogenetic Nomenclature, ISCN2013”, a total of 60 dividing phases were counted using AI chromosome image analysis system, 20 karyotypes were analyzed and repeated for 3 times. On the other hand, 3 mL of parents’ peripheral blood were collected with heparin anticoagulation, and inoculated in the phytohemagglutinin (PHA) culture medium for further karyotype analysis.

### Array-based comparative genomic hybridization (aCGH)

10 mL amniotic fluid was extracted, centrifuged at 2500 g and washed by PBS for 2 times. Genomic DNA were extracted using QIAamp® DNA Mini Kit (Qiagen, Germany). The concentration and purity of DNA were detected by NanoDrop-1000 (Thermo, USA) (optical density ratio 1.7 < 260/280 < 2.0, 2.0 < 260/ 230 < 2.5), then DNA samples were diluted to 50 ng/μl. 4x44K Human Genome CGH Microarray (Agilent, USA) was prepared. The average distance of the chip probe is 43 KB (the average distance of the control gene probe was 24 KB). The fetus and control samples were marked, hybridized and washed according to the manufacturer’s instruction. Data scanning and extraction were performed by SureScan Microarray Scanner (Agilent, USA) and its supporting software. For data analysis, CytoGenomics software (Agilent) and related databases (UCSC, DECIPHER, ISCA, etc.) were used.

## Results

### Noninvasive prenatal testing

**Fig. 1:**
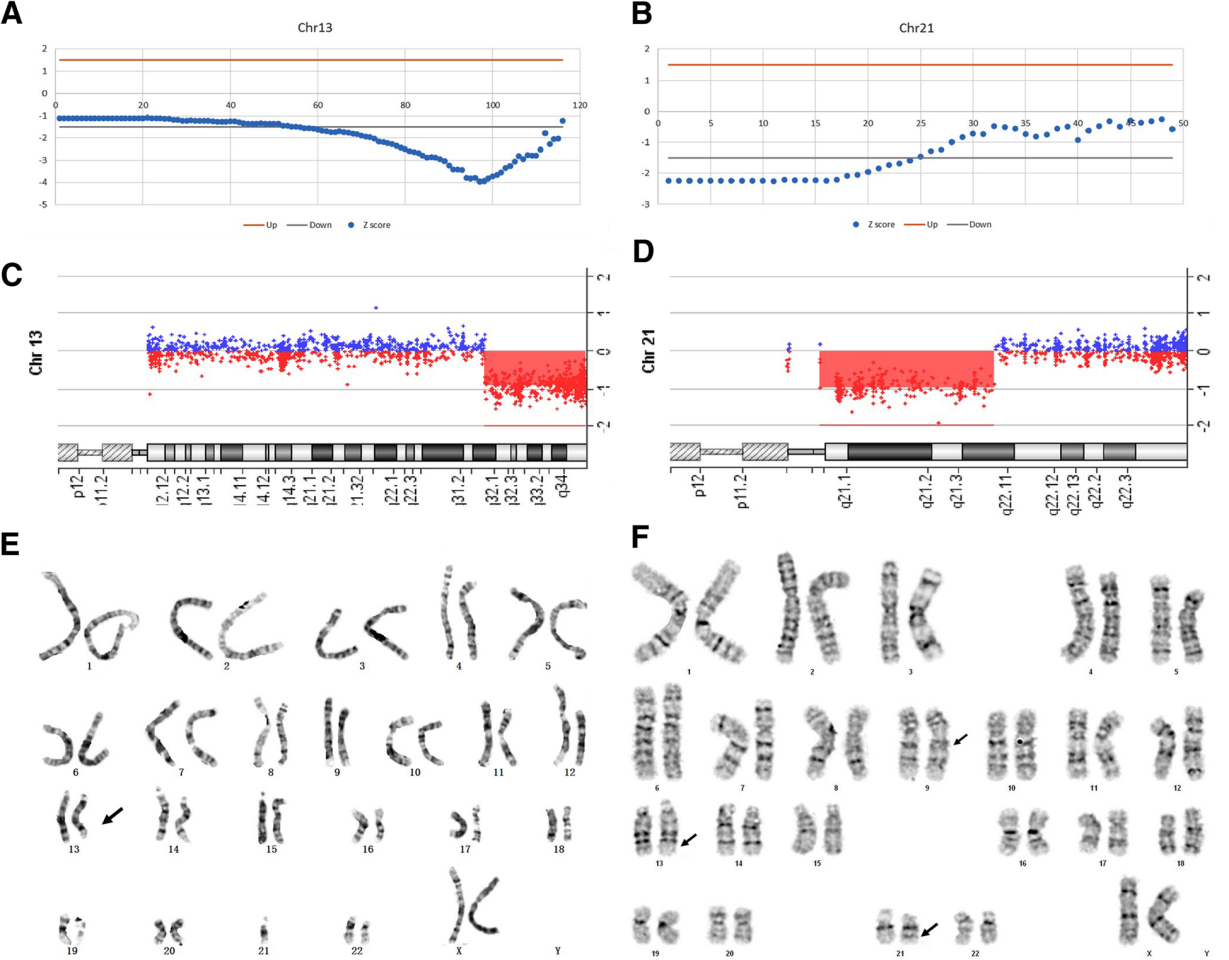
Genetic testing of fetus and her parents. **a** a partial deletion in chromosome 13 was detected by NIPT; **b** a partial deletion in chromosome 21 was detected by NIPT; **c** about 22.0 Mb deletions in q31.3-q34 of chromosome 13 was validated by aCGH; **d** about 16.0 Mb deletions in q11.1-q21.3 of chromosome 21 was validated by aCGH; **e** the fetal karyotype was 45,XX, der(13),-21; **f** the maternal karyotype was 46,XX,inv(9)(p12q13),t(13;21)(q31.3;q21.3)

The NIPT results showed that the Z score of chromosome 21 and 13 were −8.20 and −3.09 respectively, suggesting that deletions of fetal DNA fragments may occur in chromosome 21 and 13. Then, the method for detection of fetal chromosomal deletions/duplications of >10 Mb reported by Chen et al. was applied [7], and 20 Mb deletion in the end of the long arm in chromosome 13 and 15 Mb deletion in the proximal of the long arm in chromosome 21 were detected (Fig. 1a, b).

### Array-based comparative genomic hybridization

To further verify the results of NIPT, amniocentesis was conducted at 20 weeks of gestation. aCGH was performed using fetal amniotic fluid cells, and the results showed about 22.0 Mb deletion in 13q31.3 - 13q34 (93072289–115092576) and 16.3 Mb deletion in 21q11.1 - 21q21.3 (13926078–30198760),which were consistent with the results of NIPT.

### Chromosome karyotype analysis

Karyotype analysis of amniotic fluid showed chromosome structural abnormalities (45,XX,der(13),-21) (Fig. 1e), although the precise chromosomal translocation section was unknown. Combining the results of NIPT, aCGH, and karyotype analysis, the clinical diagnosis of fetal karyotype was 45, XX, der(13)t(13;21)(q31.3; q21.3), −21. Moreover, we also analyzed the chromosome karyotypes of the parents, the maternal karyotype 46,XX,inv(9)(p12q13),t(13;21)(q31.3;q21.3) showed balanced translocation of chromosomes 13 and 21 (Fig. 1f), while the paternal karyotype revealed no obvious abnormality.

## Discussion

The discovery of cell-free fetal DNA(cffDNA) in maternal peripheral blood induced a revolution in prenatal screening, and has greatly promoted the development of NIPT [8, 9]. In this study, we successfully detected complex deletions in chromosomes 13 and 21 in a fetus using NIPT, and further located the specific deletion region precisely using aCGH. According to the chromosome karyotypes of the parents and fetus, we concluded that the complex deletions in the fetus was due to the balanced translocation of maternal chromosomes 13 and 21.

After detecting complex deletions in chromosomes 13 and 21 by NIPT, a comprehensive ultrasound examination of the fetus was performed at 22 weeks of gestation, and the ultrasound examination revealed multiple anomalies including cranial structural abnormalities (lateral ventricle dilatation, cavity of septum pellucidum not shown, slightly higher third ventricle), callosal agenesis, slightly enhanced echo of left eye crystalline lens and hydramnion. These clinical abnormalities were consistent with genetic testing results, therefore, we recommended the pregnant women to terminate pregnancy after genetic counseling.

At present, NIPT has been widely used in the detection of fetal chromosome aneuploidy, and it has become a routine clinical test for the screening of trisomy 21,18 and 13 [1–3]. However, it is still a challenge to detect subchromosomal abnormality by NIPT [4]. A study from the Jensen lab showed that they successfully detected about 3 Mb deletions in 22q11.2. However, to achieve the positive result, relatively high coverage was required with approximately 200 million reads per sample [10]. Similarly, Yu et al. used locally weighted scatterplot smoothing (LOESS) for GC correction (median counts 144,000,000 per sample) and they required 3 consecutive bins having |Z | > 3 to identify a CNV [11]. With the challenge, NIPT is constantly improved. Straver et al. reported a method to detect aberrations down to 20 Mb at relatively shallow sequencing coverage (0.15–1.66×). However, an attempt to further increase detection resolution resulted in increased number of false positives [12]. Another study by Chen et al. proposed a binary segmentation and dynamic threshold strategy to detect CNVs > 10 Mb using low-coverage sequencing data [7]. Recently, Rampasek et al. presented a probabilistic method for detecting fetal CNVs and the authors estimated that CNVs bigger than 400 kb could be detected at 90 % sensitivity if the fetal fraction is sufficiently high (13 %) [13, 14]. Using the new bioinformatics analysis algorithm, Zhao et al. reported that the whole genome subchromosome abnormality was detected in low coverage (0.2×), including microdeletions, microduplications and copy number variations [4]. This broadened the clinical application category of NIPT, and provided more genetic information in the condition of low coverage and cost. In this study, we effectively detect subchromosomal abnormality in low coverage by the Ion proton platform based on semiconductor sequencing and its optimized bioinformatics analysis algorithm by DaAn Gene limited company.

## Conclusions

In a word, NIPT has the advantages of noninvasive, early and high throughput, as well as high accuracy, it was not only suitable for the detection of the traditional chromosome aneuploidy, but also for complex fetal subchromosome structure aberrations and copy number variations. With the development and improvement of the technology, NIPT can play a greater role in the detection of more complex diseases in the future.

### Consent

This study was approved by the institutional ethics committee of Nanjing Medical University Affiliated Suzhou Hospital. Written informed consent was obtained from the patient before the study.
